# Supplementary material for: Greater Height Is Associated with a Larger Carotid Lumen Diameter
Source: Medicines (Basel). 2019 May 14;6(2):57. doi: 10.3390/medicines6020057 (PMC6631842; doi:10.3390/medicines6020057)
Supplement: Supplementary file 1 [file medicines-06-00057-s001.pdf]

Supplementary Materials: Greater Height Is Associated with a Larger Carotid Lumen Diameter

Phoenix Hwaung, Moonseong Heo, Brianna Bourgeois, Samantha Kennedy, John Shepherd and Steven B. Heymsfield

Table S1. Common carotid artery diameter (CCAD) and intima-media thickness (IMT) core prediction models (n = 231).

	ß	SE	R^2
**Model 3 CCAD** (mm)	0.77		0.20‡
Intercept	-0.258	0.89	
Height (cm)	0.029‡	0.005	
Age (years)	0.006*	0.003	
BMI (kg/m²)	0.036‡	0.007	
**Model 4 IMT** (mm)	0.18		0.24‡
Intercept	0.25‡	0.06	
Age (years)	0.005‡	0.0007	
BMI (kg/m²)	0.005†	0.002	

Both models are statistically significant at $p < 0.001$. *, $p < 0.05$; †, p < 0.01; ‡, p < 0.001. BMI, body mass index; SE, standard error.

Table S2. Common carotid artery diameter (CCAD) and intima-media thickness (IMT) core prediction models replacing BMI with %fat as a Covariate (n = 231).

	ß	SE	R^2
**Model 5 CCAD** (mm)	0.77		0.20‡
Intercept	-0.72	1.29	
Height (cm)	0.032‡	0.008	
%fat	0.035‡	0.007	
Sex (0, F; 1, M)	0.368*	0.172	
**Model 6 IMT** (mm)	0.18		0.23‡
Intercept	0.26	0.07	
Age (cm)	0.005‡	0.007	
%fat	0.004*	0.002	
Sex (0, F; 1, M)	0.07*	0.03	

Both models are statistically significant at $p < 0.001$. *, $p < 0.05$; ‡, $p < 0.001$. F, female; M, male; SE, standard error.

Table S3. Common carotid diameter (CCAD) and intima-media thickness (IMT) prediction models including lipid analyses (n = 203).

	ß	SE	R^2
**Model 7 CCAD** (mm)	0.76		0.27‡
Intercept	-0.367	0.957	
Height (cm)	0.03‡	0.005	
Age (years)	0.009†	0.003	
BMI (kg/m^2)	0.04‡	0.008	
LDL (mg/dL)	-0.005*	0.002	
**Model 8 CCAD** (mm)	0.76		0.25‡
Intercept	-0.132	1.001	
Height (cm)	0.03‡	0.005	
Age (years)	0.009*	0.003	
BMI (kg/m^2)	0.04‡	0.008	
TC (mg/dL)	-0.003*	0.001	
**Model 9 CCAD** (mm)	0.77		0.23‡
Intercept	-0.872	1.373	
Height (cm)	0.035‡	0.008	
Sex (0, F; 1, M)	0.401*	0.181	
%Fat	0.037‡	0.008	
LDL (mg/dL)	-0.004*	0.002	

Both models are statistically significant at $p < 0.001$. *, $p < 0.05$; †, $p < 0.01$; ‡, $p < 0.001$. BMI, body mass index; F, female; LDL, low-density lipoprotein cholesterol; M, male; SE, standard error; TC, total cholesterol.

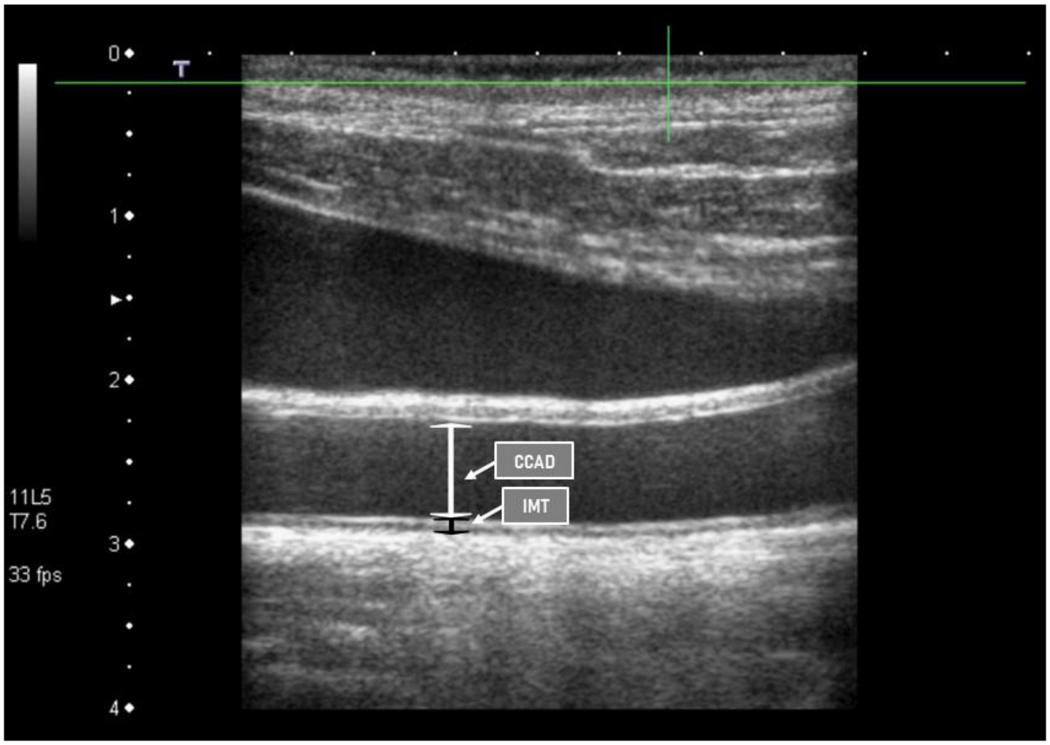

Figure S1. Ultrasound scan with common carotid artery diameter **(**CCAD) and intima-media thickness (IMT).
